# Pennsylvania State Dental Society

**Published:** 1885-10

**Authors:** William H. Trueman

**Affiliations:** Philadelphia


### PENNSYLVANIA STATE DENTAL SOCIETY.

SEVENTEENTH ANNUAL SESSION, HELD AT CRESSON, JULY 28, 29, AND 30, 1885.

REPORTED FOR THE INDEPENDENT PRACTITIONER BY WILLIAM H. TRUEMAN, PHILADELPHIA.

(Concluded from page 491.)

### TUESDAY, JULY 28—EVENING SESSION.

The session was opened by the reading of a paper entitled "Teachings of Experience," by Theodore F. Chupein, D. D. S., of Philadelphia.

The Doctor, after noting the various methods and theories that had from time to time been recognized by the profession as correct practice, gave the conclusions which he had reached as the result of thirty-three years' active practice, and the reasons which had led him to approve and adopt the methods which he advocated.

He said: At one time every effort was made to preserve the first molars, but gradually the profession has been forced to recognize the fact that the saving of these teeth was not always the best that could be done. Little by little we saw that in many cases the advantage afforded the other teeth by their removal was far greater than any evil likely to result from their loss.

When the first molars show signs of decay early in life, or where the teeth are large and the jaws small, their extraction at the proper age gives the remainder of the teeth more room for development, especially favoring the bicuspids, as well as affording room for the third molars. Not only is the third molar endangered when presenting in a crowded jaw, but the distal approximal surface of the second molar is frequently injured by the close contact, in a position where it is exceedingly difficult to properly fill.

He did not give any specific age for the extraction of these teeth, nor yet advise it as a rule without exception. The time of eruption is not always the same; there are certain individual peculiarities, certain textures of teeth, as well as other considerations, that influence our judgment, and which must be considered in deciding when to extract.

When he decides upon the loss of the first molars, he endeavors to preserve them by means of temporary fillings until there are indications that the second molars are at hand and nearly ready to erupt. By extracting them at this time, the tilting of the second molars will be prevented, as they will naturally fall nearly into the space formerly occupied by the first molars. The bicuspids usually separate slightly, thereby relieving the approximal surfaces of close, hard contact, a condition so fruitful of decay.

On the other hand, should the character of the first molars be good, the jaws large, and should there exist indications that the texture of the teeth will improve as the child advances in age, he would prefer to retain them, and risk losing the third molar by extraction as soon as it is sufficiently erupted, if the condition of the denture requires it. He considered it far better to retain only twenty-eight teeth than to endeavor to save the full complement and risk all. He also referred to the many cases of facial neuralgia, so hard to diagnose, which are caused by the growth of a third molar, unable to erupt on account of the crowded condition of the jaw.

He said that he had settled down to the conviction that gutta-percha is the best material with which to fill nearly all approximal cavities in nearly all classes of teeth. up to the twentieth or twenty-fifth year. Gold may be admissible upon the masticating surfaces, but tin or amalgam can be placed there with equal if not better effect, up to the ages indicated. He noted the fact that in many cases the gutta-percha would require repeated renewals, but this is so easily done that he thought it better to submit to that rather than to a large, wearisome, and expensive operation with gold, that would fail almost invariably in the course of a few years, through recurrence of decay. The fillings can be renewed; teeth irreparably decayed cannot.

He spoke of the practice of filling cavities at the cervical margins with gutta-percha, and completing the operation with gold, amalgam, or some one of the cements, and disapproved of it. Those which he had seen had not impressed him favorably. There was a look about them as if the harder material had pressed upon the gutta-percha and caused it to bulge or protrude. It may be admissible to use it in some cases on the floor of the cavity, to prevent thermal shock, but where possible he preferred to use one of the cements, such as the oxy-chloride, or the phosphate of zinc, or one of the non-irritant class. When it is used at the cervical margin, he recommends that it be beveled off to an extremely thin edge, so that the harder material used will completely cover it. In no case should the permanency of the filling depend upon the gutta-percha; it should be securely anchored independently of it.

He regarded with but little favor contour fillings in the bicuspids and molars made entirely with cohesive gold. When they are done on teeth narrow at the neck, or, in other words, "fan-shaped," they may be successful; in other cases decay will recur in spite of the most careful finishing and the best knuckling of gold against gold. He advocated filling the cervical portion of such cases with non-cohesive gold, or amalgam, or tin, and finishing with cohesive gold. There seems to be a therapeutic attribute in tin or amalgam not possessed by cohesive gold.

He severely condemned the practice of removing good, old-fashioned, non-cohesive gold fillings, to reinsert in their stead nice, smooth, brightly-polished, cohesive gold fillings. He had known this to be done when the old fillings were faithfully preserving the teeth in which they were placed. He regarded such practice as dictated by a spirit of egotism, looking with contempt upon the efforts of the past, rather than a spirit of honesty and justice.

He regards the zinc oxy-chloride and the zinc phosphate filling material as serviceable in lessening the time consumed in inserting large operations with gold, the larger part of the cavity being filled with the cement, and gold afterwards anchored in and built over it. They are especially useful when used in this way in devitalized teeth. Used alone, they are serviceable only upon the masticating surfaces.

He has been very successful in pulp capping in cases of recent exposure, but equally unsuccessful where there was evidence that the exposure had existed for some time. In cases where the pulp bled profusely, he was invariably successful. In cases where he found the pulp exposed after cleaning the cavity of decay, and there was little or no pain during excavation, he usually applied the arsenical paste to complete devitalization. In all cases where there was the smallest hope of success, he recommended and practiced pulp capping.

He feared that in the operation of root-filling he would be deemed an "old fogy," for despite a trial of all, or nearly all, the methods and materials recommended, he still adhered to, and practiced this operation with the use of cotton. He preferred to use it in small pieces, about the size of the head of a large pin ; used in this way it was less liable to clog.

For regulating teeth he had found the various forms of the "Coffin" spring plate invaluable, and in all cases where they could be applied he had used them exclusively.

The discussion which followed the reading of the paper was mainly upon the use of the Coffin plate in regulating.

*Dr. S. H. Guilford*—Stated (referring to the Coffin plate) that, in cases where the teeth lean so that the point of the spring has a tendency to slip off, he overcame the difficulty by fitting around the tooth a thin platina band, and cementing it in place with phosphate of zinc. In cases where he preferred not to carry the plate over the molars, he makes the points that pass between the teeth quite long, so that they will pass well into the inter-space. As the jaw expands, the plate has a tendency to loosen. This he corrects by warming the points and bending them down. In lower plates designed to expand the arch, he does not think it needful to cover the molars and bicuspids; there is no tendency in the plate to jump out of place, as is the case with an upper plate. That is prevented by the shape of the lingual surfaces of the lower molars. It is only necessary to let the plate pass over the crowns of the back teeth at one or two points, so as to prevent the plate pressing into the gum.

*Dr. C. J. Essig*—Makes use of the elasticity of vulcanized rubber to press out the anterior teeth. He makes a vulcanite plate with a stud back of the tooth to be moved, and through this he passes a screw, with the point pressing against the tooth. The object of the screw is not to move the tooth by the pressure it exerts, but simply to follow the tooth up; as the screw is tightened the rubber stud is slightly bent back, the elasticity of the stud moving the tooth. A little care is needed in constructing the plate, not to vulcanize it too hard, and not to make the stud so thick as to be rigid.

*Dr. Darby*—Suggested that care was needed in using the split plate in the case of young patients, and not to use much force, for fear of separating the palatal bones. He related a case where this had occurred, but fortunately it was noticed in time to prevent serious injury. The suture had opened from just inside the centrals to the soft palate.

Those who took part in the discussion had found the "Coffin"

plate very satisfactory. Some had difficulty in using the split form on account of the two halves twisting when it was pulled apart; when this has taken place the fit of the plate is materially impaired. It was suggested that it was not necessary to make a new plate when the springs needed changing; the old ones could be cut out and new ones revulcanized in the old plate.

*Dr. Pierce*—Suggested a simple method he had used with satisfactory results to bring out the upper centrals or laterals in young patients, when he was fortunate enough to see the irregularity just as the teeth had erupted sufficiently to slightly overlap, in cases where the upper front teeth articulated inside the lower teeth. He tied one end of a silk or gilling-twine ligature to the lower central or lateral, or included several of the teeth in a loop, and tied the other end to one of the molars of the same side. This was done on both sides of the mouth, the two ligatures forming a triangle. He then passed a thread over both of them, and by forming a knot drew them together a little distance back of the front teeth. This tightened the ligatures and drew the teeth in so far that in the course of from twenty-four to forty-eight hours the lower teeth were found articulating inside the upper ones. The ligature was then removed, the lower teeth acting upon the upper ones, gradually forcing them into position. He had used this method in a large number of cases, and found it by far the most rapid and satisfactory of any he had tried.

The examining board reported that three candidates had appeared before them, and having passed a satisfactory examination had been granted certificates of qualification.

WEDNESDAY, JULY 29—MORNING SESSION.

Dr. Louis Jack, of Philadelphia, explained at length his method of using the various forms of the matrix which he has introduced to facilitate the filling of approximal surfaces, and especially to produce contour work, with the least waste of time and labor in introducing the gold and polishing the filling. He illustrated his remarks by the use of diagrams. We did not note anything which has not been previously published, and as the illustrations are necessary to a proper understanding of his remarks, and of his method of using and adjusting the matrix and forming the filling, we have not attempted to report them. In forming the cavity, Dr.

Jack opens from the crown in operating upon the approximal superior surfaces of the molar and bicuspid teeth, cutting away largely in order to gain ready access to the cavity of decay, and to thoroughly pack the gold against the edges of the cavity when the matrix is in place. He forms well defined dovetails on each side of the cavity, so as to securely hold the filling. When the cavity is ready for filling, a horizontal or cross-section of it closely resembles a horse-shoe in shape, or perhaps more nearly the capital letter G.

*Dr. Guilford*—Disapproves of this, contending that by so shaping the cavity the sides are unnecessarily weakened, and liable to break away, and also, that it brings a much larger surface of the filling to the edge of the crown, where it is more exposed to the force of occlusion, which tends to tear it from its position. He prefers to cut away no more than is absolutely necessary to make the walls straight or parallel. In order to make the filling secure, he carries it farther over the crown and makes the anchorage remote from the approximal surface, where it does not weaken the tooth. A cross section of a cavity prepared as Dr. Guilford suggests will be somewhat V-shaped, the anchorage being made at the point of the V. This not only conserves the strength of the tooth, but also materially lessens the strain upon the filling, as a large part of the force of occlusion is received in a direct line with the axis of the tooth, which tends to hold it in place rather than to dislodge it.

He considered the loop matrix far preferable, as a rule, to those advocated by Dr. Jack. It is more readily applied and more readily manipulated, as it does not depend upon the adjoining tooth for support, and permits, he thinks, of more accurate and secure adjustment. In finishing approximal contour fillings he first shapes the cervical margin with a thin, delicate instrument, shaped like a straight gum lancet, and very sharp. After this he uses thin bladed files, some with the blade bent at a right angle, and finishes with polishing tape.

*Dr. Darby*—Finds great satisfaction in using matrices made of steel. He takes an old clock spring (he prefers the Seth Thomas make on account of their excellent quality) and rolls it down in his rolls to the thickness desired. Before rolling, he carefully anneals it so as to make it as soft as possible, to avoid injury to the rolls. Some are quite thick, others as thin as the steel can be made.

For some cases he prefers brass, or phosphor-bronze. These metals are readily swaged to any shape required, and on this account, in some cases, are preferable to steel. This form of matrix is easily made. The metal is cut to the required shape, one or more little ears being formed on the upper edge and bent at right angles, so as to rest upon the adjoining tooth to prevent the matrix from slipping into the gum, and, at the same time, to assist in holding it securely. After smoothing the edges, they are bent with the pliers so as to snugly embrace the tooth. They are so curved as to give the filling the desired contour. He is very careful to secure a close and accurate fit at the cervical edge of the cavity, so as to leave as little finishing at this point as possible. He has discarded wooden wedges to secure them in place on account of their liability to break when made thin, as they must be in many cases, and also because they often work loose and are difficult to readjust. He uses in their place thin, spring-tempered steel wedges, made to screw into a steel handle. While in the handle they are readily forced into position, and when in place the steel handle is easily unscrewed and removed. He also exhibited a neat arrangement for the same purpose, consisting of two steel wedges so arranged that by turning a nut they can be made to approach each other. After the matrix is adjusted, this is placed in position; the wedges fit between the teeth, and by turning the nut are made to hold the matrix quite firmly. If, during the operation, it should become loose, a slight movement of the nut readjusts it. The arrangement is quite small, and when in position does not seem to be in the way. The matrices, with the little ears to assist in holding them secure, are the suggestion of Dr. T. A. Woodward, of Philadelphia; the screw arrangement and the steel wedges are the invention of Dr. Darby.

*Dr. W. B. Miller*, of Altoona—Exhibited a simple matrix device which he has invented, that seems quite practical and will no doubt prove useful. He has fitted to each flange of a broad rubber dam clamp (Dr. Tees' pattern) a small screw with a flat head. To this is fitted various shaped matrices made with a slotted flange to rest upon the flange of the clamp, the slot passing under the head of the screw. The matrices may be used upon either side of the clamp. It is used as follows : The slot of the matrix is passed under the screw and the clamp adjusted upon the tooth. The matrix is now adjusted by bending, etc., being removed and replaced if necessary. When satisfactory as to shape and position, the screw is tightened, and will hold it sufficiently firm for most plastic operations. When greater firmness is desired, a fine wire is passed around the matrix and the tooth, and the ends twisted together. This will hold it firmly enough for all operations. It has the advantage of not depending upon an adjoining tooth for support.

AFTERNOON SESSION, JULY 29.

The afternoon session was entirely taken up by the election of officers, with the following result :

*President*—Dr. J. W. Rhone, Bellefonte.

*First Vice-President*—Dr. Louis Jack, Philadelphia.

*Second Vice-President*—Dr. J. P. Thompson, Johnstown.

*Recording Secretary*—Dr. E. P. Kremer, Lebanon.

*Assistant Secretary*—Dr. W. B. Miller, Altoona.

*Corresponding Secretary*—Dr. W. H. Fundenberg, Pittsburg.

*Treasurer*—Dr. J. C. M. Hamilton, Tyrone.

*Board of Censors*—Dr. C. J. Essig, Chairman; Dr. A. Boice, Secretary; Drs. C. S. Beck, Gale French, W. H. Trueman. (The latter has since resigned.)

*State Examining Board*—Drs. Jack and Beck were elected to succeed Drs. Gerhart and C. N. Pierce. (It is quite probable that the election of these gentlemen may be questioned. There is a resolution of the Society which provides that "there shall be one, and only one, from the faculty of each college represented in the Society, on the Board."* The election of these gentlemen, depriving the Pennsylvania College of Dental Surgery of representation on the Board, was in direct violation of this, and accompanied by a scene of disorder, of which no society can afford to be proud.)

Cresson was selected as the next place of meeting.

EVENING SESSION, JULY 29.

Dr. William H. Trueman read a paper upon "The Importance of Accurate Impressions in Mounting Pivot Teeth and other like Operations."

---

* See transactions Pennsylvania State Dental Society, 1878, page 79.

The paper reflected the doctor's experience as a mechanical dentist, and his observations in the workroom of those who adopted that branch as their calling.

He expressed surprise that, while the importance of correct impressions for all forms of artificial dentures was fully recognized, so many seemed to think that an impression by which to mount a pivot tooth was a matter of trifling moment. He stated that it was a common occurrence for a cast from a rudely taken impression in wax, on which the outline of the root was but faintly shown, to be brought to a dental laboratory with instructions to construct upon it a pivot arrangement, and that the mechanic was expected to drill the opening for the pivot with no other guide as to its position and direction than a slight depression marking the opening to the pulp canal. If the operator placed a piece of wood in the canal before the impression was taken, he seemed to think any inaccuracy in the finished work was inexcusable. He had noticed that those who worked thus carelessly usually had but little success with pivot teeth mounted on gold, and were prone to abandon that method for others less reliable and less durable.

For the operation to be a success, the root should be fully prepared, and the pivot that is to be used in the finished piece carefully fitted into the root, and in the position it is to occupy when the impression is taken. The impression should always be taken in plaster, so as to avoid the possibility of any unseen change taking place when the impression is taken from the mouth. In these cases he discarded the impression cup and built the plaster into the space over the root and around the adjoining teeth with a spatula. In this way a practically perfect impression and cast can be obtained. He called attention to the point that, while an ordinary denture is fitted to a yielding surface, a pivot plate is fitted to a hard, unyielding root, hence the necessity of greater, not less, accuracy.

The fact that these fixtures are held in place with cement that will fill any little inaccuracy under the plate does not lessen the importance of a good fit. Unless the cement is amalgam, the permanency of the operation and its cleanliness depend upon the plate fitting closely all around the edges, so as to protect the cement from wear or disintegration. The same accuracy in the model is called for in bridge work, in the so-called Richmond crowns, and other methods of replacing lost teeth, or repairing the ravages of decay by other methods than by filling.

He thought the extra time required to obtain a good cast well spent. It is all returned by the time saved when the fixture is inserted in the mouth, while the satisfaction of a good piece of work well done continues as interest upon the investment as long as the work lasts. He thought that many dentists who do not do their own mechanical work do not appreciate the extra labor and the constant annoyance an imperfect model makes in the laboratory, neither do they realize that it is absolutely impossible to construct a well-fitting piece of work upon a defective cast.

The discussion which followed the reading of the paper brought out no points of interest.

Dr. Magill, of Erie, read a paper upon "The Law of 1876, with Supplement," in which he called attention to the difficulty of so framing a law that the objects desired can be at once accomplished. The law of 1876 was the best that could then be obtained, although defective in many points. The supplement was intended to remedy this, but had failed to do so. It was difficult to define, under the law, what really constituted a proper qualification for the practice of dentistry. He could see no way out of it but to fall in line with other States and require a diploma from a recognized dental college in all cases. He did not consider the law a failure, neither did he consider the time and labor which had been spent upon it lost. The profession had been gaining experience in a new field, and a public sentiment had been growing up in the community that he had no doubt would in time enable us to accomplish all we desired. Reforms that work real good move slow, but they move sure.

The reading of the paper was followed by a general discussion on the subject of dental law, which ended in the presentation and adoption of a resolution instructing the Committee on Legislative Action to endeavor to have the law of this State amended, so that a diploma from a dental college shall be required in all cases as a prerequisite to practice.

The Committee on Enforcement of the Dental Law made their report. There has been no case before them during the past year. Several cases have been reported, but they had learned from experience to take no action unless they were sure that reliable evidence could be secured, and this they found very difficult to obtain.

They had to depend upon those living in the immediate neighborhood of the delinquent to secure testimony, and while many were ready to make complaint to the committee, they were not so ready when asked to secure evidence upon which the committee could work. The courts require *facts*, and pay no attention to hear-say, and these facts were far more difficult to obtain than those who had no experience in the matter would suppose.

THURSDAY MORNING, JULY 30.

Dr. C. S. Beck, of Wilkesbarre, spoke at length upon "Treatment of Children's Teeth." The following is a *resumé* of his remarks:

The question has often been asked, at what age should children be brought to the dental office for examination? He usually answered it by saying about the third or fourth year. By early attention, not only is the child saved a great deal of suffering and the probability of a good and regular denture increased, but by judicious management on the part of the dentist the natural timidity of the child in the dental chair is overcome. The good effect of this may last through life. He desired to impress most earnestly the importance of always being truthful. The most important point in managing children is to secure their confidence. To this end he always selected the most simple cavities for filling during the first visit, those he was sure would give the least pain and discomfort. While he endeavored to clean and excavate as perfectly as the case demanded, if there was any degree of sensitiveness, he preferred, if necessary, to allow a little of the disintegrated dentine to remain, rather than by attempting to thoroughly prepare the cavity to give the child that intense horror of dental operations which so many patients possess, and which in many cases may be traced to the injudicious manner in which the first dentistry they had submitted to was performed. He preferred to do the best he could at the time—to preserve the teeth until the child is older and better able to bear and to appreciate the importance of the operations.

He prefers to treat abscess of the temporary teeth, rather than to bridge over the pulp chamber and drill a vent; there is something very repugnant to him in the latter operation. After cleaning the pulp chamber and canals as well as he is able, he uses a weak solution of corrosive sublimate, two grains to the ounce of water, using it quite freely, by working it in with a lock of cotton, or a roll of bibulous paper, or by means of the syringe. He uses this on account of its potency in destroying bacteria, and has had the happiest results from its employment. In other cases he fills the pulp chamber with warm water by means of the syringe, and then applies pure carbolic acid by means of rolls of bibulous paper or cotton, and endeavors to make it reach thoroughly to all parts of the pulp chamber and canals, and to pass through the fistulous opening if one exists. By these means he thoroughly washes out the parts and renders them clean and sweet.

In cases where there is a fungus growth at the mouth of the fistula, he cuts that off with the scissors, and immediately applies carbolic acid, full strength. He does not expect by these means to cure the abscess, but he makes them far less painful and more comfortable in every way.

When the tooth is ready to fill he uses gutta-percha in the pulp chamber, preferring the base-plate variety. Sometimes he dips it into chloroform just before passing it into the cavity. The outer surface being softened, it seems to make a more perfect filling. He does not attempt to fill the canals. They are usually so minute in the molars that he allows them to take care of themselves, while the larger canals of the anterior teeth suggest caution, lest the filling be forced through the foramen.

He constantly endeavors to make the visits of his little patients as pleasant as possible. He feels that by so doing he is not only conferring upon them a present benefit, but is laying the foundation for a confidence in, and appreciation of, dental operations, that will last as long as they live.

The discussion added but little, practically, to what Dr. Beck had said.

Dr. Templeton, of Pittsburg, read a paper, entitled "Too Little of a Good Thing." (See page 517.)

The new President, Dr. J. W. Rhone, of Bellefonte, was now introduced, and, on taking the chair, appointed the following committees:

*Executive Committee*—Drs. W. B. Miller, C. S. Beck, C. J. Essig, W. H. Fundenberg, M. B. Lowrie.

*Publication Committee*—Drs. W. H. Trueman, W. B. Miller, E. P. Kremer, C. N. Pierce, E. T. Darby, S. H. Guilford.

*Enforcement of Dental Laws*—Drs. W. E. Magill, G. L. Robb, Jos. R. C. Ward.

*Committee on Legislative Action*—Drs. C. N. Pierce, H. Gerhart, J. A. Todd, W. E. Magill, G. W. Klump.

Adjourned to meet at Cresson the last Tuesday of July, 1886.

